# Immune responses to vaginal candidiasis in African women: A scoping review of cytokine profiles, T-cell activation, and gene expression

**DOI:** 10.1371/journal.pone.0322072

**Published:** 2025-04-23

**Authors:** Bwambale Jonani, Bwire Roman Herman, Joel Fredrick Arturo, Richard Kwizera, Gerald Mboowa, Felix Bongomin

**Affiliations:** 1 Department of Immunology and Molecular Biology, School of Biomedical Sciences, College of Health Sciences, Makerere University, Kampala, Uganda; 2 Laboratory Department, Sebbi Hospital, Wakiso, Uganda; 3 Department of Research, Infectious Diseases Institute, College of Health Sciences, Makerere University, Kampala, Uganda; 4 The African Centre of Excellence in Bioinformatics and Data-Intensive Sciences, Infectious Diseases Institute, Makerere University, Kampala, Uganda; 5 Department of Medical Microbiology and Immunology, Faculty of Medicine, Gulu University, Gulu, Uganda; Universidade dos Açores Departamento de Biologia: Universidade dos Acores Departamento de Biologia, PORTUGAL

## Abstract

**Background:**

Recent immunological studies of vaginal candidiasis in African populations have revealed complex host‒pathogen interactions with implications for therapeutic development and HIV acquisition risk.

**Objective:**

This scoping review synthesized evidence from Uganda, Zambia, South Africa, and Kenya between 2020 and 2024, focusing on immune responses, cellular dynamics, and tissue effects due vulvovaginal candidiasis.

**Results:**

Analysis revealed a coordinated inflammatory response marked by elevated levels of the proinflammatory cytokines IL-1β and IL-6 and increased chemokine IL-8-mediated immune cell recruitment. Compared with those in control individuals, distinct T-cell population patterns in colonized individuals show reduced Th17-like CD4+ T-cell activation, with concurrent increases in Th1/Th2-enriched CD4+ T cells. Molecular analysis revealed that 162 differentially expressed genes were involved primarily in neutrophil-mediated immunity and cytokine signaling pathways. Despite robust immune activation, tissue integrity remained intact, accompanied by elevated antimicrobial peptides SLPI and BD-2. Notably, Candida-colonized individuals presented reduced frequencies of HIV target cells (CCR5 + HLA-DR + CD4 + T cells).

**Conclusion:**

These findings advance our understanding of population-specific immune responses to vaginal candidiasis and identify promising therapeutic targets, highlighting the need for longitudinal studies to characterize vulvovaginal candidiasis immunopathogenesis fully in African populations.

## Introduction

Vulvovaginal candidiasis (VVC), an inflammatory yeast infection of the vagina and/or vulva, is caused by fungal pathogens of the genera Candida, Nakaseomyces, Pichia, and other new genera, including former Candida species such as *Debaryomyces, Clavispora, Kluyveromyces, Meyerozyma, Wickerhamomyces, and Yarrowia* [1].When a patient experiences at least four episodes within 12 months, with two episodes confirmed by microscopy or culture when symptomatic, the condition is classified as recurrent vulvovaginal candidiasis (RVVC) [2]. Of particular concern is the high prevalence of VVC in Africa, where it affects 33% of women, with pregnant women being six times more likely to develop the infection than nonpregnant women are [3].

Despite this significant prevalence, our understanding of how African women of reproductive age respond immunologically to Candida colonization or infection remains limited. The general immune response to Candida species is well documented, beginning with barrier functions provided by epithelial cells [4]. These cells express various pathogen recognition receptors (PRRs), including Toll-like receptors (TLRs 2, 4, and 9), cysteine-like receptors (CLRs such as dectin 1, dectin 2, DC-SIGN, mincle, and mannose binding lectin), and galectin family proteins [5,6]. These PRRs recognize specific pathogen-associated molecular patterns (PAMPs) on fungi, primarily β-1,3-glucans, chitin, and mannans, initiating immune responses that vary depending on the cell types involved [5].

The immune response cascade involves several cellular components and pathways. Monocytes, macrophages, neutrophils, and epithelial cells directly combat fungal pathogens through phagocytosis [7]. Upon encountering fungal pathogens, dendritic cells mature and promote the differentiation of naïve T cells into effector T cells [4]. The binding of PAMPs to TLRs and CLRs activates multiple downstream pathways, including the SYK-CARD9 and RAF pathways, ultimately leading to the production of protective factors, such as defensins, chemokines, cytokines, reactive oxygen species, and indoleamine 2,3-dioxygenase [7].

A significant challenge in managing vaginal Candida infections is the high prevalence of asymptomatic cases, which necessitates routine screening and early detection to prevent complications and reduce transmission [8,9]. This silent nature of infections highlights the need for biomarkers that can predict the onset, replication, or resolution of vaginal Candida infections. Furthermore, given that the epidemiology and immune responses to Candida infections vary significantly by region and race owing to genetic, environmental, and microbial factors [10,11], African populations may exhibit unique immune response patterns, whose knowledge remains very limited.

This scoping review aimed to consolidate and provide a comprehensive landscape of immune markers related to vaginal candidiasis in African women. By synthesizing the available evidence, we sought to provide insights into immune mechanisms, identify research gaps, and suggest public health and clinical implications specific to this population.

## Materials and methods

### Study design, inclusion criteria, and exclusion criteria

This qualitative scoping review was performed according to the Preferred Reporting Items for Systematic Reviews and Meta-analysis (PRISMA) checklist **(SI File).** The systematic review protocol was registered with PROSPERO (CRD42024554917). Studies were included if they focused on immune markers in African women with vaginal candidiasis or colonization. Initially, we excluded studies whose geographical region was not Africa and then restricted the study participants to African women. The outcome measure of the study had to be cytokines, chemokines, the cellular response, or differential gene expression due to vulvovaginal candidiasis.

### The search strategy

On 4^th^ May 2024, Jonani Bwambale (JB), Bwire Roman Herman (BRH) and Arturo Fredrick Joel (AFJ) independently searched 4 databases: PubMed, Scopus, African Journals online, and Google Scholar. The search terms included “vaginal candidiasis,” “cytokines,” “T cells,” “gene expression,” “Africa,” and “Candida immune response.” The Scopus database was accessed through Harzing’s Publish and Perish software.

### Review of studies

The researchers JB, AFJ, and BRH independently reviewed the records via the following steps: All records were imported to the Zotero citations manager (Version 6.0.36). The search filter “Africa” and all names of the 54 African countries were used to select studies from the African region. Then, “cytokine,” “chemokine,” “CD4,” “CD4+”, “SNP,” “DGE,” “neutrophil,” “inflammation,” and “helper cell” filters were applied to studies from Africa. Titles and abstracts of records retrieved at this stage were screened to identify those that addressed the immune response to either vulvovaginal candidiasis or recurrent vulvovaginal candidiasis. The outputs of this screening process were compared with each reviewer, and disagreements were resolved by consensus. Two researchers, JB and BRH, independently screened the full-text articles for inclusion. In case of any disagreements, a consensus was reached on inclusion/exclusion by discussion and, where necessary, consultation with the AFJ.

### Critical appraisal

We assessed the risk of bias using the Newcastle Ottawa Scale for cohort studies. The appraisal tool addressed three main domains: (1) Bias arising from the selection of participants, (2) bias from comparability of cohorts based on the design or analysis, and (3) Bias in measurement of the outcome measures. Two review authors; BJ and HRB independently applied the tool to each study and recorded supporting information and justification of the score awarded to each domain. These risks of bias scores were independently reviewed by AFJ, with resulting discrepancies resolved through discussion by all three reviewers. The overall risk of bias was determined using the rating: 0–4 points for critical risk of bias, 5–6 for moderate risk of bias, and 7–9 for low risk of bias. The studies were then classified as unsatisfactory, satisfactory, and very good studies respectively.

### Data summary

We designed a data extraction Excel form in which two reviewers, JB and BRH, extracted data from the eligible studies (**S2 File).** The extracted data were compared, and any discrepancies were resolved through discussion and a joint review of the paper or the affected section. The extracted data included the title, DOI, authors, year of publication, study population, age range of participants, country where the study was conducted, sample size, study design, inclusion and exclusion criteria, immune markers studied, laboratory methods used, statistics used, main findings, ethical considerations, and limitations of the study. The extracted data were further double-checked by BF, RK, and GM for accuracy.

### Ethics approval and consent to participate

This systematic review used published data and did not require ethical approval.

## Results

### Search results

**Fig 1:**
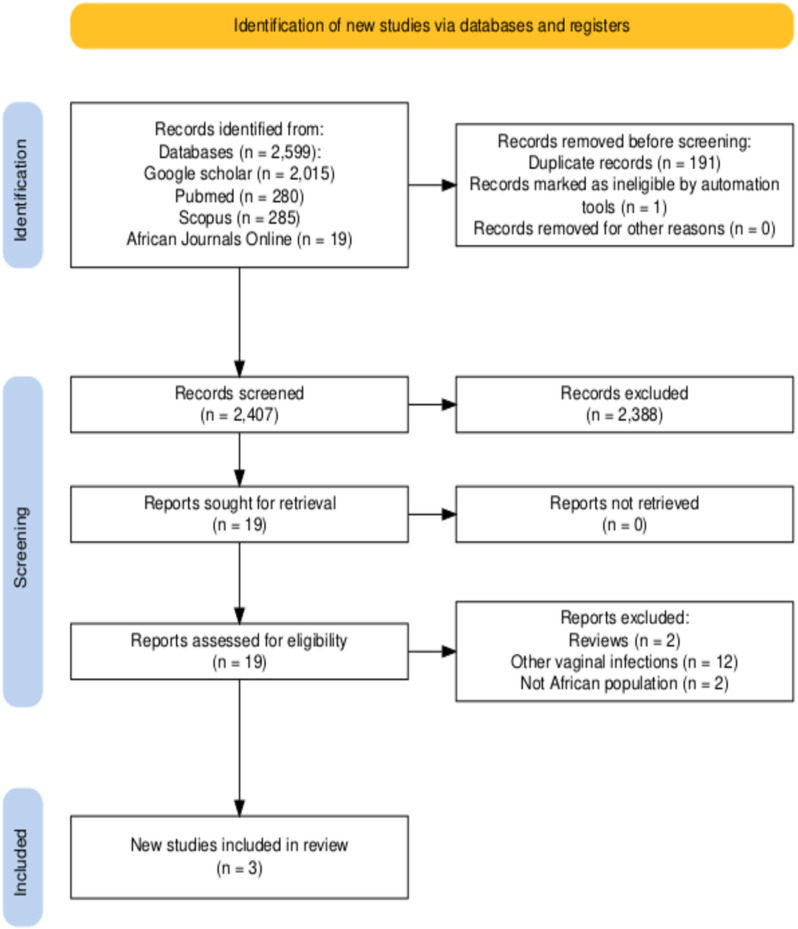
PRISMA flow diagram showing the study selection process for a scoping review on the immunology of vulvovaginal infections in Africa.

We found 2,599 records from the database searches. After removing duplicated articles, we screened 2,407 records, from which we reviewed 19 full-text documents and ultimately included 3 papers [12–14], as shown in Fig 1.

## Summary of studies

We included three studies with 1,172 participants from four African countries: South Africa, Kenya, Zambia, and Uganda Table 1

**Table 1:** Cytokines, chemokines, activated immune cells and differentially expressed genes measured in each study.

Author, year, reference	Study Design (Sample size)	Type of sample used	Country	Cytokine and Chemokines Measured	Cells and Genes
Fichorova., et al 2020 [12]	Prospective cohort study (934)	Cervical swabs and secretions	Uganda and Zambia	Interleukin (IL)-1β, IL-6, IL-8 (CXCL-8), IL-1 receptor antagonist (IL1RA), RANTES (CCL-5), macrophage inflammatory protein (MIP)-3α (CCL-20), vascular endothelial factor (VEGF), and soluble intercellular adhesion molecule (ICAM)-1 (CD54), soluble leukocyte protease inhibitor (SLPI) and BD-2	
Happel et al., 202 [13]	Randomized control trial (130)	Vaginal secretions and Cervicoctyobrush samples	South Africa	Cytokines associated with Th17 cell differentiation (IL-1β, IL-6, TNF-α, IL-23, and IL-33), cytokines produced by Th17 cells (IL-17A, IL-17F, IL-21, and IL-22), and cytokines related to Th17 cell regulation (IL-25, IL-31, IFNγ, and soluble CD40 ligand)	CD31 CD41 T cells, CD31 CD41 T cells
**Hasselrot et al., 2024** [14]	Retrospective cohort (108)	Ectocervical biopsies from the superior part of the ectocervix	Kenya		CD11c+, CD14+, Monocytes, Macrophages

### Critical appraisal of individual sources of evidence

A summary of these assessments is provided in Table 2, showing a text summary for each of the three individual segments of Newcastle Ottawa Scale, against which each record was assessed.

**Table 2:** Risk of bias assessment for each study and the three domains adopted from the Newcastle Ottawa scale.

Author, year, reference	Bias from Selection of participants	Bias from Comparability	Bias from measuring Outcome	Overall risk of bias	Classification
Hasselrot et al., 2024 [14]	Moderate risk	Low risk	Moderate risk	Moderate risk	Satisfactory
Happel et al., 2022 [13]	Low risk	Low risk	Low risk	Low risk	Good study
Fichorova., et al 2020 [12]	Low risk	Low risk	Low risk	Low risk	Good study

### Proinflammatory signaling

A coordinated inflammatory response has been observed in vaginal candidiasis-positive women compared with candidiasis-free controls in Uganda and Zambia, where the proinflammatory cytokines IL-1β (2.75 pg/mg, p = 0.0075) and IL-6 (11.77 pg/mg, p = .0039) initiate an immune cascade, triggering chemokine IL-8 (974.12 pg/mg, p = 0.0002)-mediated immune cell recruitment [12]. This inflammatory milieu is further modulated by increased RANTES (2.10 pg/mg, p = 0.0295) and MIP-3α (330.97 pg/mg, p = 0.0005) signaling, which facilitates immune cell communication. Similar inflammatory patterns have been observed in South African women, where those with hyphal forms present significantly elevated IL-22 levels (12.79 pg/mL, IQR: 6.80–40.78) compared with those without hyphae (6.38 pg/mL, IQR: 4.62–11.07, p = 0.034), alongside increased IL-17A and IL-17F expression [13].

### Immune cell response

**Fig 2:**
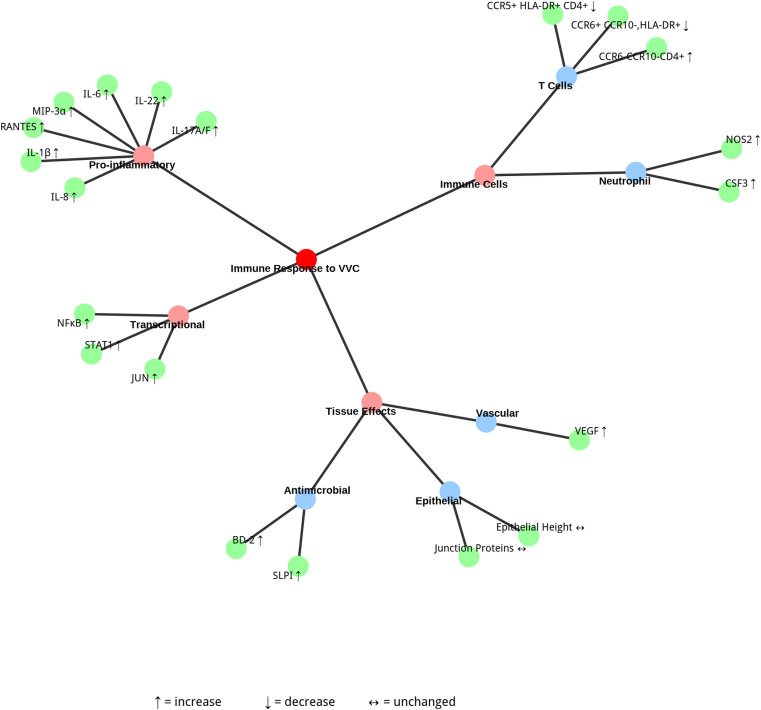
A concept map showing cytokines, T-cell subsets, and gene expression responses linked to Candida infection.

Complex immunological patterns associated with persistent, asymptomatic Candida colonization, particularly in the presence of hyphal forms, have been shown to be characterized by distinct T-cell population dynamics in the cervicovaginal environment in Kenyan women, Fig 2. The enhanced Th17 response is accompanied by a distinctive T-cell profile in which colonized individuals present reduced activation of Th17-like CD4+ T cells (CCR6+ CCR10−, HLA-DR+) at 30.10% (IQR: 16.58–42.78) compared with that of noncolonized participants (47.25%, IQR: 23.12–52.15, p = 0.018), while simultaneously exhibiting increased frequencies of Th1/Th2-enriched CCR6− CCR10− CD4+ T cells (44.70%, IQR: 34.95–53.67 vs 36.50%, IQR: 30.75–48.00, p = 0.041). Notably, the presence of HIV target cells, represented by activated CCR5 + HLA-DR + CD4+ T cells, was lower in colonized individuals (14.10%, IQR: 8.79–21.40) than in noncolonized individuals (20.00%, IQR: 10.80–30.10, p = 0.047) [14].

### Transcriptional regulation

Molecular analysis of vaginal Candida infection in Kenyan sex workers revealed significant transcriptional changes, with 162 differentially expressed genes (DEGs) comprising 147 upregulated and 15 downregulated genes [14]. These genes are linked to immune activation pathways, particularly neutrophil-mediated immunity, with enrichment in pathways such as TNF-alpha signaling via NF-κB and IL-6/JAK/STAT3 signaling. Pathway enrichment analysis highlighted immune activation pathways such as neutrophil-mediated immunity and cytokine‒cytokine receptor interactions, with the transcription factors RELA (log2FC 2.15, p < 0.05), REL (log2FC 2.10, p < 0.05), NFKB1 (log2FC 1.95, p < 0.05), JUN (log2FC 2.20, p < 0.05), and STAT1 (log2FC 2.05, p < 0.05) identified as central regulators of these responses. The significantly upregulated genes include IL1B (log2FC 2.42, p < 0.0001), IL6 (log2FC 2.67, p < 0.05), NOS2 (log2FC 2.70, p < 0.05), and CSF3 (log2FC 2.67, p = 0.412).

### Tissue effects

The tissue response to vulvovaginal candidiasis is characterized by enhanced vascularization through VEGF (415.08 pg/mg, p = 0.0226) upregulation, along with concurrent increases in the antimicrobial peptides SLPI (22092 pg/mg, p = <.0001) and BD-2 (660.26 pg/mg, p = 0.0016) [12]. Despite this robust immune activation, analysis of tissue integrity parameters in the Kenyan cohort revealed no significant differences between groups in terms of epithelial height, epithelial junction protein (EJP) coverage, or CD4+ cell distribution, suggesting maintenance of tissue integrity despite the active immune response [14].

## Discussion

This review of the inflammatory responses to vaginal candidiasis in African women reveals an intriguing and unexpected relationship between fungal colonization and HIV susceptibility. Our findings revealed a coordinated immune response characterized by elevated levels of proinflammatory cytokines (IL-1β, IL-6, and IL-8) alongside a surprising reduction in the number of HIV target cells (CCR5+ HLA-DR+ CD4+ T cells) in colonized individuals. This observation challenges the traditional paradigm that increased inflammation necessarily correlates with increased HIV susceptibility.

The distinctive pattern of immune activation observed in African women, characterized by reduced Th17-like CD4+ T-cell activation alongside increased Th1/Th2-enriched populations, appears to be further modulated by combined oral contraceptive (COC) use. This builds upon previous work [15–17], and extends our understanding by demonstrating specific impacts of hormonal contraception on immune modulation in African populations. The observed upregulation of inflammatory cytokines among COC users aligns with studies from other geographical regions [18,19], but notably, our findings suggest a more complex immune environment than previously recognized.

Our review has significant implications for treating vulvovaginal candidiasis. The cytokine patterns we observe, specifically the increased levels of IL-1β, IL-6, and IL-8, indicate that additional therapies targeting proinflammatory pathways may be beneficial in reducing symptoms and preventing recurring infections. While the decrease in HIV target cells (CCR5+ HLA-DR+ CD4+ T cells) in individuals with Candida colonization is a promising finding, it currently serves as a potential avenue for therapeutic and preventive strategies, emphasizing the need for further evidence to support these approaches

The reduced frequency of HIV target cells in colonized individuals (14.10% vs 20.00%, p = 0.047) represents a particularly important finding that warrants further investigation. This observation suggests that while Candida colonization induces robust inflammatory responses, it may simultaneously create an environment less conducive to HIV infection through mechanisms that remain to be fully elucidated. Understanding the interaction between vaginal candida colonization/infection and the expression of HIV target cells in endemic areas, such as African approaches to HIV prevention.

Thus, large-scale longitudinal studies across diverse African populations are needed to establish whether this reduction in HIV target cells is maintained over time and correlates with actual HIV acquisition risk. There is also a need to further explore the interplay between the vaginal microbiota, Candida species, and mucosal immunity to help explain how specific Candida species colonization influences HIV target cell populations in the vaginal area. Additionally, there is a need to investigate how different COC formulations affect vaginal immune responses to Candida species and inform more tailored contraceptive recommendations.

Despite limitations in the current evidence, including the small number of studies included and variations in the study designs, our findings reveal important insights into the immunology of vaginal candidiasis. A meta-analysis would have strengthened statistical insights. However, the limited number of eligible studies (n = 3) and heterogeneity in outcome measures precluded meaningful quantitative synthesis. While the influence of specific Candida strains and their interactions with the -microbiome of the vaginal environment remain unclear, the observed relationship between colonization and reduction of HIV target cells suggests that host‒pathogen interactions in the vaginal environment are more sophisticated than previously recognized. This discovery opens new avenues for understanding HIV disease susceptibility in African populations.

## Conclusion

The unexpected finding of reduced HIV target cells in Candida-colonized individuals, despite elevated inflammatory markers, reveals a complex and potentially protective host‒pathogen interaction in African women that challenges the traditional understanding of vaginal immunity. Large-scale longitudinal studies across diverse African populations are urgently needed to further define the interactive mechanisms between Candida species and HIV target cells in the vaginal environment. These studies could revolutionize our approach to improving women’s health in HIV-endemic regions by informing the development of interventions that maintain beneficial immune responses while managing candidiasis.

## Supporting information

S1 File Preferred Reporting Items for Systematic reviews and Meta-Analyses extension for Scoping Reviews (PRISMA-ScR) Checklist.
(PDF)

S2 File Data extracted from the 3 studies included in this systematic review. (XLSX)

## Abbreviations:

VVC: Vulvovaginal Candidiasis
rVVC: Recurrent Vulvovaginal Candidiasis
BV: Bacterial Vaginosis
STI: Sexually Transmitted Infections
PAMP: Pathogen-Associated Molecular Patterns
PRRs: Pathogen Recognition Receptors
TLRs: Toll-Like Receptors
NLRs: NOD-Like Receptors
RCT: Randomized Control Trial
RLRs: RIG-I-like receptors
PMNs: Polymorphonuclear cells
PRISMA: Preferred Reporting Items for Systematic reviews and Meta-Analyses
rRNA: Ribosomal Ribonucleic acid
PCR: Polymerase Chain Reaction
SNP: Single-Nucleotide Polymorphism
SLPI: Secretory Leukocyte Protease Inhibitor
BD-2 Beta: Defensin 2
RANTES: Regulated upon Activation, Normal T-Cell Expressed and Secreted
DGE: Differential Gene Expression
qPCR: Quantitative PCR
CVL: Cervicovaginal Lavage
cpn60: Chaperonin 60
JB: Jonani Bwambale
BRH: Bwire Roman Herman
AFT: Arturo Fredrick Joel
GM: Gerald Mboowa
